# Editorial

**Published:** 1899-04

**Authors:** 


### HOMŒOPATHIC PHYSICIAN,

A MONTHLY JOURNAL OF

HOMŒOPATHIC MATERIA MEDICA AND CLINICAL MEDICINE.

If our school ever gives up the strict inductive method of Hahnemann, we are lost, and deserve only to be mentioned as a caricature in the history of medicine."—CONSTANTINE HERING.

Vol. XIX. APRIL, 1899. No. 4.

### EDITORIAL.

NOTICE TO SUBSCRIBERS.—It has been impossible for the editor of this journal to find time to prepare the editorials for which some of our readers have lately been making inquiries.

The demands upon the editor, by reason of his being in active practice, have been doubled and even quadrupled during the past winter by the outbreak of the grippe, followed a little later by the wonderful epidemic of typhoid fever through which this great city has been passing. To these demands upon his time, which have continued far into the night and have even prevented his getting to bed at all, the editor has to attend to a considerable correspondence, and in addition prepare this journal every month for the readers. As a consequence much of the correspondence yet remains unanswered, and as the profession knows, the journal itself is much behind time in making its appearance. Under such circumstances it is impossible to find time to prepare the notes of Dr. Lippe's lectures for publication.

The subscribers are therefore requested to bear with us for the delinquencies which are referred to, and to look forward to the time when sufficient leisure may be had to make good these deficiencies.

Meanwhile, those readers who are also members of The International Hahnemannian Association are once more reminded that the meeting of that organization will soon take place, and they are urged to make strong efforts to be present at the meeting, and in the interval, to prepare contributions to the work of the society by the recital of clinical cases and the preparation of essays upon medical subjects from the homœopathic point of view.

Those who are not members should make application for admission to our society, and prepare papers that will be of assistance in our work and become a valuable addition to our archives. These archives are published as books and should be possessed by all good homœopathists, and carefully read and pondered, and their contents stored in the memory for practical use when making prescriptions.

We hope to see an excellent attendance at the meeting.

At present we may say that the meeting will probably be held at The International Hotel, Niagara Falls, June 27th.

---

INOCULATION AND VIVISECTION.—In Germany a new practice has arisen of performing experimental inoculations of Dr. Koch's Tuberculin upon new-born children in hospitals. Not only Tuberculin, but various other kinds of bacteriological cultures, the malignant virus of black smallpox pus from abcesses and the like have been injected. Dr. Epstein, professor of children's diseases in Prague, infected young children with round worms for experiment. Some of these experiments are detailed in *The Deutche Medical Wochenschaft*, of February 19th, 1899. Dr. R. E. Dudgeon, of London, has attacked and exposed these dreadful procedures in the name of science. His article is a terrible indictment of the whole practice of inoculation and vivisection, and an arraignment of their authors. It is published in *The Abolitionist*, a new anti-vivisection journal published in London.

